# Breaking the annotation barrier: An initial subcellular localization atlas of *Giardia*’s hypothetical and conserved hypothetical proteins provides a resource for functional discovery

**DOI:** 10.1091/mbc.E25-12-0590

**Published:** 2026-04-10

**Authors:** Kari D. Hagen, Alexander J. Zerkle, Satvik Arani, Tiffany Chase, Michael J. Cipriano, Matthew P. Hirakawa, Jonathan K. Pham, David J. Woessner, Christopher Nosala, Shane G. McInally, Nicholas A. Hilton, Joseph A. Williams, Kristopher Nguyen, Gregory T. Walker, Lorita Boghospor, Allen B. Tu, Andrew Bluhm, Sharon Jan, Katie Chun, Gary Du, Albert C. Sek, Jacqueline Booker, Scott C. Dawson

**Affiliations:** ^a^Department of Microbiology and Molecular Genetics, University of California, Davis, Davis, CA 95616

## Abstract

*Giardia intestinalis* is a globally prevalent cause of waterborne diarrheal disease, yet about 40% of its proteome remains functionally uncharacterized due to the lack of conserved homologous proteins and limited experimental validation of protein function. To begin addressing this gap, we created a large-scale subcellular localization resource by fluorescently tagging and imaging 608 *Giardia* proteins (12% of the proteome) expressed in live cells from native promoters. This dataset includes 240 hypothetical proteins, 215 domain-family proteins (including ankyrin repeat and NEK kinase families), 171 diplomonad- or *Giardia*-specific proteins, 69 conserved eukaryotic proteins, and 77 proteins with known functions that were previously unlocalized. Imaging revealed localization to cytoskeletal and *Giardia*-specific organelles (eight flagella, the ventral disk, and the median body), along with novel components of the plasma membrane and endomembrane systems. Integrating localization data with domain architecture, homology, and *Giardia*-specific Gene Ontology terms, we produced a “localization-informed” gene annotation with a standardized, structured nomenclature. This resource provides the largest experimentally validated functional annotation of the *Giardia* proteome to date, linking predicted gene models to cellular structures, creating testable hypotheses for protein function and establishing a durable framework for future studies of cell biology, pathogenesis, and eukaryotic evolution in this deeply divergent diplomonad lineage.

## Abstract (executive summary)

- Defines the spatial context and experimentally validates hundreds of predicted lineage-specific and conserved eukaryotic hypothetical proteins, linking predicted gene models to cellular structures and generating testable hypotheses for protein function.
- Highlights previously undiscovered molecular innovations that underlie *Giardia* cytoskeletal, nuclear, and membrane biology.
- Creates and implements a standardized, “localization-informed” and structured nomenclature for gene annotation that will serve as a foundation for future *Giardia* functional genomic and genetic analyses.

## INTRODUCTION

*Giardia intestinalis* is a zoonotic intestinal protozoan and the causative agent of giardiasis—globally one of the most prevalent protistan parasitic diseases (Hotez, 2008). The *G. intestinalis* (syn. *G. lamblia*, *G. duodenalis*) genome sequencing effort began in 2000 with the WB strain (assemblage A, clone C6), culminating in the first draft genome publication in 2007. It revealed a compact 11.7 Mbp genome encoding 6470 predicted ORFs with minimal intron content (Morrison *et al.*, 2007). Subsequent *G. intestinalis* genomes, including the GS isolate (assemblage B) in 2009 and the pig-derived P15 isolate (assemblage E) in 2010, expanded the comparative framework and highlighted extensive gene-family variation between strains, particularly among variant-specific surface proteins (VSP) (Franzén *et al.*, 2009; Jerlstrom-Hultqvist *et al.*, 2010). The 2020 long-read assembly of the *G. intestinalis* WB isolate, sequenced using PacBio RS II long reads (∼3.6 Gbp; ∼286 × coverage) and polished with Illumina short reads (∼100 × coverage), generated a near-complete, chromosome-scale reference genome comprising five chromosomes (∼12.6 Mb) with only four internal gaps (Xu *et al.*, 2020). The updated annotation included extensive revision of gene models, with 626 gene start sites corrected and numerous small open reading frames (ORFs) removed or merged, resulting in a final set of 4963 protein-coding genes, 306 pseudogenes, eight cis-spliced introns, and five trans-spliced introns. These improvements substantially enhanced both protein domain annotation and overall gene annotation quality, providing the first high-contiguity reference for *Giardia* and enabling more accurate downstream comparative and experimental analyses.

Most recently, additional high-contiguity assemblies from diverse isolates have continued to refine our knowledge of *Giardia*’s genomic diversity (Klotz *et al.*, 2023; Sun *et al.*, 2025). Yet despite *Giardia’*s medical significance and the availability of comprehensive genomic, transcriptomic, and proteomic data for close to 20 years (Morrison *et al.*, 2007), our understanding of *Giardia* biology is still very limited, especially with respect to the subcellular organization and experimentally validated functions of *Giardia* proteins. The compact 12.6 Mb genome of *Giardia* with about 5000 protein-coding genes is comparable in size and content to the genome of the model yeast *Saccharomyces cerevisiae*. However, in contrast with *S. cerevisiae* and many other model organisms, nearly 42% of *Giardia* genes are annotated as “hypothetical proteins,” indicating they lack detectable homology to known proteins in other organisms (Xu *et al*., 2020).

The detection of protein homology underpins both gene annotation and inferences of protein function (Kumar, 2025), and the extensive number of uncharacterized genes in *Giardia* poses a major challenge for understanding protein function and for the accuracy or utility of predictive functional genomic approaches that underlie genome-wide gene enrichment analyses based on gene ontologies (GO) (The Gene Ontology Consortium *et al.*, 2023). In the absence of clear, full-length homology to proteins of known function, inference of protein function is unreliable or impossible, even when there is homology to a conserved protein domain. In such cases, standard pipelines can overcall predicted cellular functions or, conversely, can leave large numbers of proteins annotated as “hypothetical.” Without a homologue, there is no reference point for the biochemical role, cellular localization, interaction partners, or biological importance of a predicted protein. As a result, proteins in *Giardia* (and other phylogenetically distant non-model organisms) remain systemically under-annotated or inaccurately annotated, ultimately slowing biological discovery in this important human parasite.

In addition to numerous novel proteins, there are many *Giardia* proteins with homology only to conserved protein domains such as ankyrin repeats or NEK (NIMA-related) kinases, both of which are overrepresented in the genome. Specifically, the WB2020 reference genome annotation identified 184 NEK kinases and over 305 ankyrin repeat proteins, comprising close to 10% of the proteome (Xu *et al*, 2020). Proteins with uncharacterized or ambiguous domains can complicate the inference of cellular roles through bioinformatic analysis alone. Even when conserved domains with well-defined functions, such as ATPases or kinases, are detected, these domain annotations fail to predict substrate specificity or a functional cellular context, such as biochemical activity or subcellular localization. For instance, *Giardia*-specific transporters often exhibit low sequence similarity to characterized transporters and may have undefined substrate binding affinities (Pearson, 2015). Likewise, proteins with roles in unique aspects of *Giardia* biology, such as encystation, attachment, or the functions of its mitochondrion-related mitosomes, may be encoded by “orphan” or nonhomologous proteins that defy homology-based functional predictions.

Because predictive functional genomic approaches are insufficient for eukaryotes that are only distantly related to well-studied model organisms such as yeast, experimental strategies are essential to systematically define protein localization, dynamics, and function. In such cases, subcellular localization can provide a critical first insight into protein function. The microaerophilic *Giardia* trophozoite exhibits a uniquely complex architecture, including eight flagella, two nuclei, and *Giardia*-specific organelles, including the median body, mitosomes, and an elaborate ventral disk for attachment. The localization of proteins to *Giardia*-specific structures is not predictable from sequence similarity to characterized proteins in other organisms.

In *Giardia*, fluorescent tagging with live-cell imaging of protein localization is a powerful alternative to antibody-based immunolocalization, which is often hampered by the lack of *Giardia*-specific antibodies and by potential fixation artefacts. For example, actin localization in *Giardia* has varied dramatically depending on fixation conditions and antibody specificity, underscoring the need for direct fluorescent visualization (Paredez *et al.*, 2011). Importantly, Green Fluorescent Protein (GFP)-tagging has also helped correct earlier mislocalization and incorrect functional prediction in *Giardia*, such as in the case of the so-called “median body protein” or MBP. MBP was initially localized only to the median body using an antibody, but was later localized to the ventral disk using tagging approaches and live-cell imaging (Hagen *et al.*, 2011). Subsequent CRISPR-based gene knockout of MBP dramatically altered the form and function of the ventral disk, but not the median body (Hagen *et al.*, 2025).

Despite its tetraploid (double-diploid) genome, stable transfection systems for Assemblage A and B strains are well-established, and fluorescent protein tags such as GFP, mNeonGreen, and mCherry have facilitated real-time imaging of trophozoites under physiologically relevant conditions for over 25 years (Hagen *et al.*, 2024). Such tags enable dynamic studies of cytoskeletal behavior, flagellar beating, and mitosis, and can be combined with techniques like fluorescence recovery after photobleaching (FRAP) to quantify protein turnover (Dawson and House, 2010a). Other live tagging systems, including SNAP, Halo, and APEX2, as well as epitope tagging in fixed parasites, offer orthogonal strategies for imaging or proximity labeling (Regoes and Hehl, 2005; Ástvaldsson *et al.*, 2019). More recent advances in genetic manipulation allow more sophisticated functional genetic studies in *Giardia*, enabling experimental molecular genetic studies of protein function. Both episomal plasmids and genome integration strategies are now widely used, and CRISPR/Cas9-based methods have recently expanded the toolkit for gene knockout and knockdown (Horáčková *et al.*, 2022; Hagen *et al.*, 2024; 2025).

Here, we used a “first-pass” high-throughput approach to clone and express selected C-terminally GFP-tagged *Giardia* proteins in the active, vegetative trophozoite stage of the *Giardia* life cycle, and present an updated functional annotation of over 600 *G. intestinalis* proteins obtained after live imaging of fluorescent trophozoites. Although not exhaustive, this imaging resource includes the subcellular localizations of many poorly characterized proteins, including hypothetical proteins, NEK kinases, and ankyrin repeat proteins. Proteins with known functions in model eukaryotes served as proof of principle, ensuring that GFP-tagged proteins were properly folded and localized in *Giardia*, and that sufficient signal was produced for imaging. We used the observed localization patterns to revise existing gene annotations, yielding a “localization-informed” functional protein annotation that integrates protein and domain homology with subcellular localization. This dataset represents a major advance in protein functional annotation, covering more than 12% of the predicted *Giardia* proteome (Xu *et al.*, 2020), and complements ongoing improvements in genome assembly of diverse *Giardia* strains and other related diplomonads. Ultimately, these data provide a valuable foundation for future molecular genetic studies of *Giardia* cell biology and pathogenesis as well as for genome-wide functional genomics studies in this widespread parasitic protist and its close relatives.

## MATERIALS AND METHODS

Request a protocol through *Bio-protocol*

### *Giardia* cultivation and electroporation

All strains used in this study were derived from *Giardia lamblia* WBC6 (ATCC 50803). Frozen stocks were thawed and cultured for at least 24 to 48 h before phenotypic analysis. Routine cultivation was performed in 16 ml screw-cap tubes (BD Falcon) containing TYI-S-33 medium (Keister, 1983) supplemented with 0.05% ovine/bovine bile, 5% adult bovine serum, and 5% FBS. Cultures were incubated at 37°C without agitation. Upon reaching confluence, tubes were chilled on ice for 30 min to detach trophozoites, and 0.5 to 1 ml of the resulting suspension was inoculated into 12 ml of prewarmed fresh medium.

Electroporation of episomal plasmids was performed using previously established protocols (Hagen *et al.*, 2025). Briefly, 1 × 10⁷ trophozoites were electroporated with 20 to 40 µg of DNA using a Bio-Rad GenePulser XL and 4-mm cuvette at 375V, 700Ω, and 1000 µF. Electroporated cells were transferred to culture tubes and maintained at 37°C with medium replaced every 48 h. Following electroporation, puromycin selection began at 10 µg/ml and was increased to 50 µg/ml when tubes were ≥50% confluent. Cultures were maintained under selective pressure for at least 1 to 2 wk before cryopreservation with 9% DMSO. Alternatively, electroporated cells were immediately resuspended in 4 ml of TYI-S-33 medium and transferred to 6-well anaerobic culture plates. After 24 h, the medium was aspirated and replaced, and puromycin was added to 15 µg/ml. Plates were incubated for 7 to 14 d at 37°C in Mitsubishi AnaeroPack 2.5 L rectangular jars containing Mitsubishi AnaeroPack-Anaero Gas Generator sachets (Thermo Fisher Scientific). Medium was aspirated and replaced every 5 to 7 d. Upon visible outgrowth, puromycin was increased to 50 µg/ml, and confluent cultures were transferred to 16 ml screw-cap tubes with 12 ml medium for routine propagation.

### Construction of C-terminal GFP and mNeonGreen (mNG) fusion strains

C-terminal GFP-tagged constructs were generated using Gateway cloning as described previously (Hagen *et al*., 2011). PCR primers were designed to amplify full-length *G. lamblia* WBC6 ORFs, excluding stop codons, plus about 200 bp of upstream promoter sequence. Amplifications were performed using either *PfuTurbo* Hotstart or Easy-A High-Fidelity PCR Master Mix (Agilent). PCR products were cloned into the entry vectors pENTR/D-TOPO or pCR8/GW/TOPO (Thermo Fisher Scientific) and confirmed by Sanger sequencing. Gateway LR recombination reactions were used to clone inserts into the destination vector pcGFP1Fpac (GenBank: MH048881), compatible with *Giardia* expression (Dawson, 2010a). Recombinant constructs were verified by AscI digestion, and plasmids were prepared using the Qiagen Plasmid Plus Midi Kit. mNeonGreen fusions were cloned into the plasmid pKS_mNeonGreen-N11_PAC as described previously (McInally *et al.*, 2019).

### Live imaging of fluorescently tagged strains

For live imaging, GFP- or mNG-expressing strains were thawed and cultured in TYI-S-33 medium for at least 24 h. Cells were chilled on ice 30 min, centrifuged at 900 × *g* for 5 min, resuspended in warm medium, and seeded into MatTek dishes or black-walled 96-well glass-bottom imaging plates (Cellvis). Cells were incubated for 1 h at 37°C to promote attachment. Immediately before imaging, the culture medium was gently replaced with prewarmed 1 × HEPES-buffered saline (HBS). Additional HBS washes were applied as needed to remove loosely attached cells. Differential interference contrast (DIC) and epifluorescence images were acquired using a Leica DMI6000B microscope as described previously (Hagen *et al.*, 2025). Image processing was performed using FIJI (ImageJ v8) (Schindelin *et al.*, 2012).

### Protein localization resource folder and nomenclature

The dataset contains comprehensive imaging data for all GFP-tagged strains, including new images of strains from our initial MT cytoskeletal proteomes (Hagen *et al.*, 2011; Nosala *et al.*, 2020). The full dataset of images is available for free download at DataDryad.org: https://doi.org/10.5061/dryad.jm63xsjrn. Each strain (indicated by gene ID) has a corresponding folder with five files: a single raw (unprocessed) DIC TIFF image, a raw FITC (green) TIFF image stack, processed and cropped versions of both the DIC and FITC images, and a cropped PNG fluorescence image of one representative cell. The DIC and FITC images correspond to the same microscope field. Image processing consisted of adjustments to contrast or brightness. In addition, single FITC images were created from FITC stack files, either by choosing the most representative slice or by performing a maximum or average intensity projection of in-focus slices. Raw file names include the date the image was taken, gene ID, image number, and whether it is the DIC or FITC file. Cropped and processed files are prepended with “cropped,” and the single-cell files are prepended with “one cell.” “MAX” indicates a maximum intensity projection.

## RESULTS AND DISCUSSION

**FIGURE 1:**
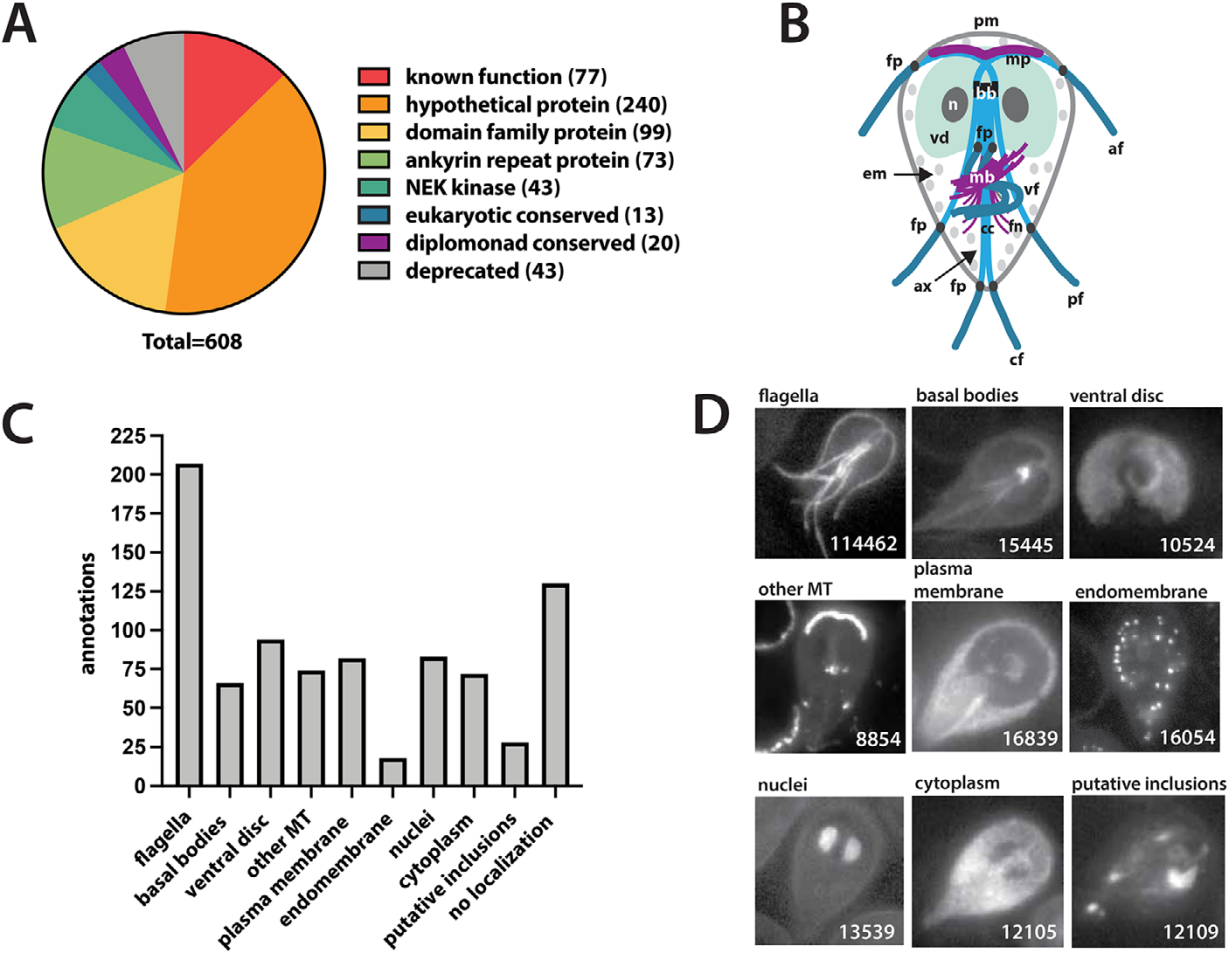
Summary of subcellular localizations and annotation categories. In (A), the 608 GFP or mNG-tagged proteins are summarized and grouped into annotation categories that include proteins with “known function” and proteins with known protein domains only; that is, overrepresented “ankyrin repeat domain” and “NEK kinase domain” proteins or other “domain family proteins.” Other categories include proteins that lack functional annotations but are conserved only in diplomonads (“diplomonad conserved”) or those that are conserved throughout diverse eukaryotes (“eukaryote conserved”). Tagged proteins lacking any homology to known proteins (“hypothetical”) are also summarized. The schematic (B) shows key subcellular features of trophozoites that were used for localization-informed annotation and for GO categorization: pm, plasma membrane; em, endomembrane system; n, nuclei; bb, basal bodies; vd, ventral disk; mb, median body; fn, funis; cc, caudal complex; mp, marginal plate; fp, flagellar pores. Four flagellar pairs (af, anterior; pf, posteriolateral; cf, caudal; vf, ventral) have both membrane-bound portions (dark blue) and nonmembrane-bound, cytoplasmic axoneme portions (light blue, ax). In (C), annotations are grouped by subcellular localization category, with representative images for each shown in (D). Proteins may belong to more than one subcellular localization category. “Other MT” includes cytoskeletal structures unique to *Giardia*: the marginal plate (shown in D), funis, median body, and caudal complex. “Putative inclusions” indicates putatively mislocalized proteins that form cellular aggregates or proteins associated with previously unknown *Giardia* endomembrane organelles. “No localization” indicates that no fluorescence was detected in the trophozoite stage in the tagged strain.

We analyzed 608 ORFs from *G. intestinalis* using GFP tagging to assign subcellular localization and ultimately to update protein annotations. The GFP C-terminal localization data annotation provides functional assignments for a range of previously uncharacterized *Giardia* proteins, including those lacking homology to proteins in other organisms (240 hypothetical proteins), plus proteins of unknown function that are conserved in eukaryotes (13 eukaryote conserved proteins) or only in diplomonads (20 diplomonad conserved proteins) (Figure 1A). Another 215 tagged proteins simply had a conserved protein domain (domain family proteins), such as the common ankyrin repeat proteins (73 total) or NEK kinases (43 total). We also tagged 77 proteins that were homologous to proteins with known functions but had not been previously localized in *Giardia*. A total of 45 tagged proteins from our set of 608 were labeled as “deprecated” in the revised, localization-informed annotation. The deprecated ORFs correspond to proteins tagged before the WB2020 reference genome refinement that were not included in the WB2020 genome due to updated gene models. Our localization data indicated that most tagged deprecated proteins had no localization (i.e., no GFP signal detected) or formed putative inclusions or aggregates, supporting the assertion that the earlier gene models were incorrect.

### *Giardia*-specific localization and gene annotation

To account for unique components of the *Giardia* cell in gene ontology GO annotations (The Gene Ontology Consortium *et al.*, 2023), we used previously existing GO annotations where appropriate (e.g., microtubule-associated complex [GO:0005875]) but also developed new GO terms for *Giardia*-specific organelles and structures (see Supplemental Table S1; Figure 1B). For example, new GO terms for mitosomes (GO:0032047), the ventral disk (GO:0097597), the median body (GO:0097568), and the cyst wall (GO:0097570) were used to annotate GFP-based subcellular localizations. These terms capture structural features unique to *Giardia* cell biology and are essential for functional annotation pipelines.

For summary localizations, we used six primary categories of annotation: 1) flagellar localization (membrane-bound and cytoplasmic flagellar regions as well as flagella pores and basal bodies), 2) the ventral disk and lateral crest, 3) other MT-based organelles including the median body, marginal plate, caudal complex and funis, 4) plasma membrane, endomembrane, or nuclear membrane localization, 5) nuclear localization, including nucleoli and chromosomes, and 6) cytoplasmic localization (excluding nuclei). Most localizations were to the flagella, ventral disk, or cytoplasm, with significant numbers of annotated proteins localizing to membranes, basal bodies, or nuclei (Figure 1, C and D). Large or random-sized GFP accumulations in the cytoplasm with no regular pattern and not associated with known organelles were classified as inclusions and presumed to be due to mislocalization; however, it is possible that some or many of these are not GFP-localization or aggregation artefacts but actually mark undescribed or unknown endomembrane compartments. We acknowledge this possibility with the annotation “putative inclusion.”

### Assigning subcellular localization and annotation to *Giardia* proteins of unknown function

**FIGURE 2:**
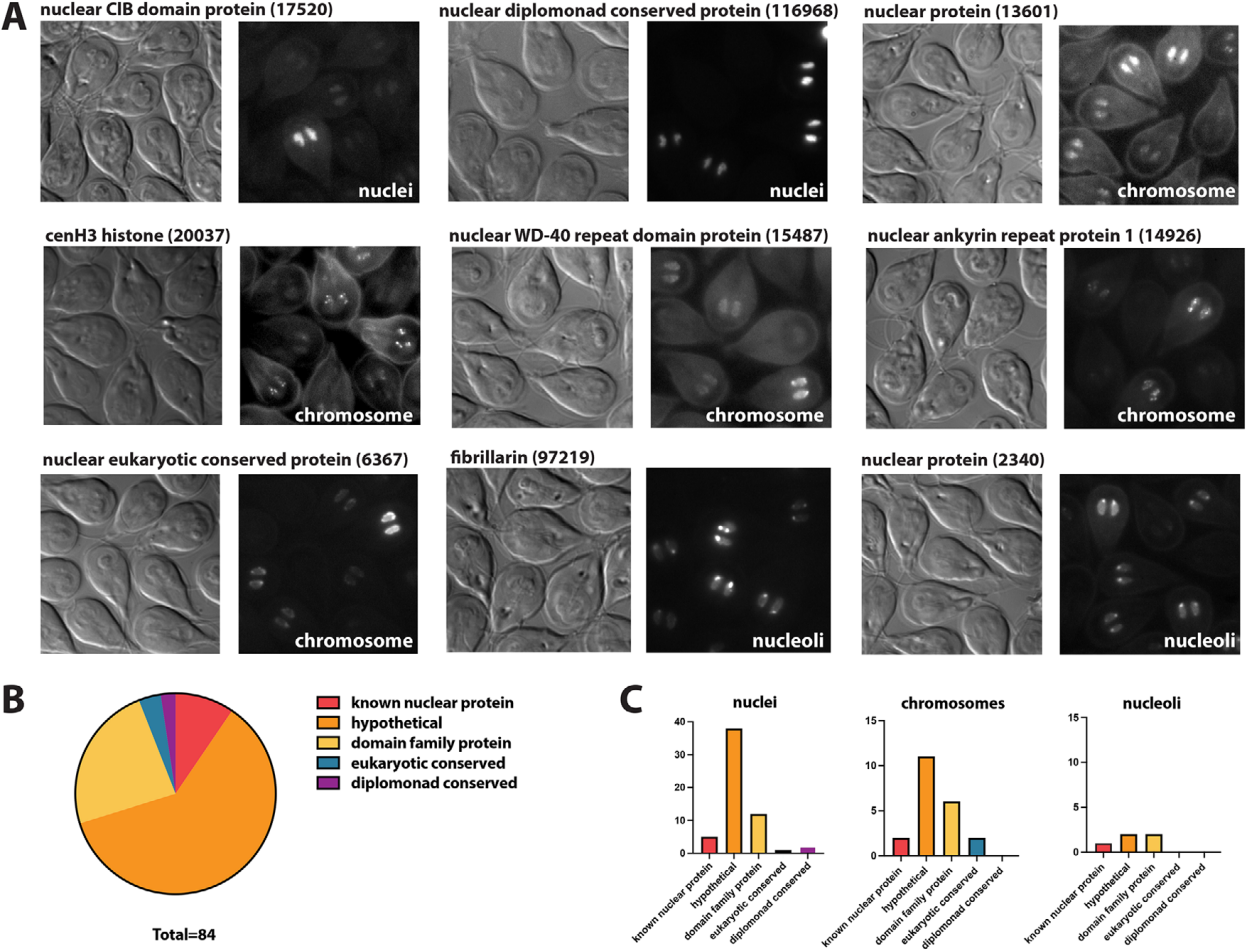
Summary localizations of nuclear, nucleolar, and chromosomal tagged proteins. Representative images are shown (A) for selected tagged proteins of the 79 total localizing to the nuclei. Corresponding GiardiaDB ORF IDs are indicated in parentheses. In (B) and (C), each of the annotation categories is summarized, with “domain family proteins” also including the ankyrin repeat proteins and NEK kinase proteins.

**FIGURE 3:**
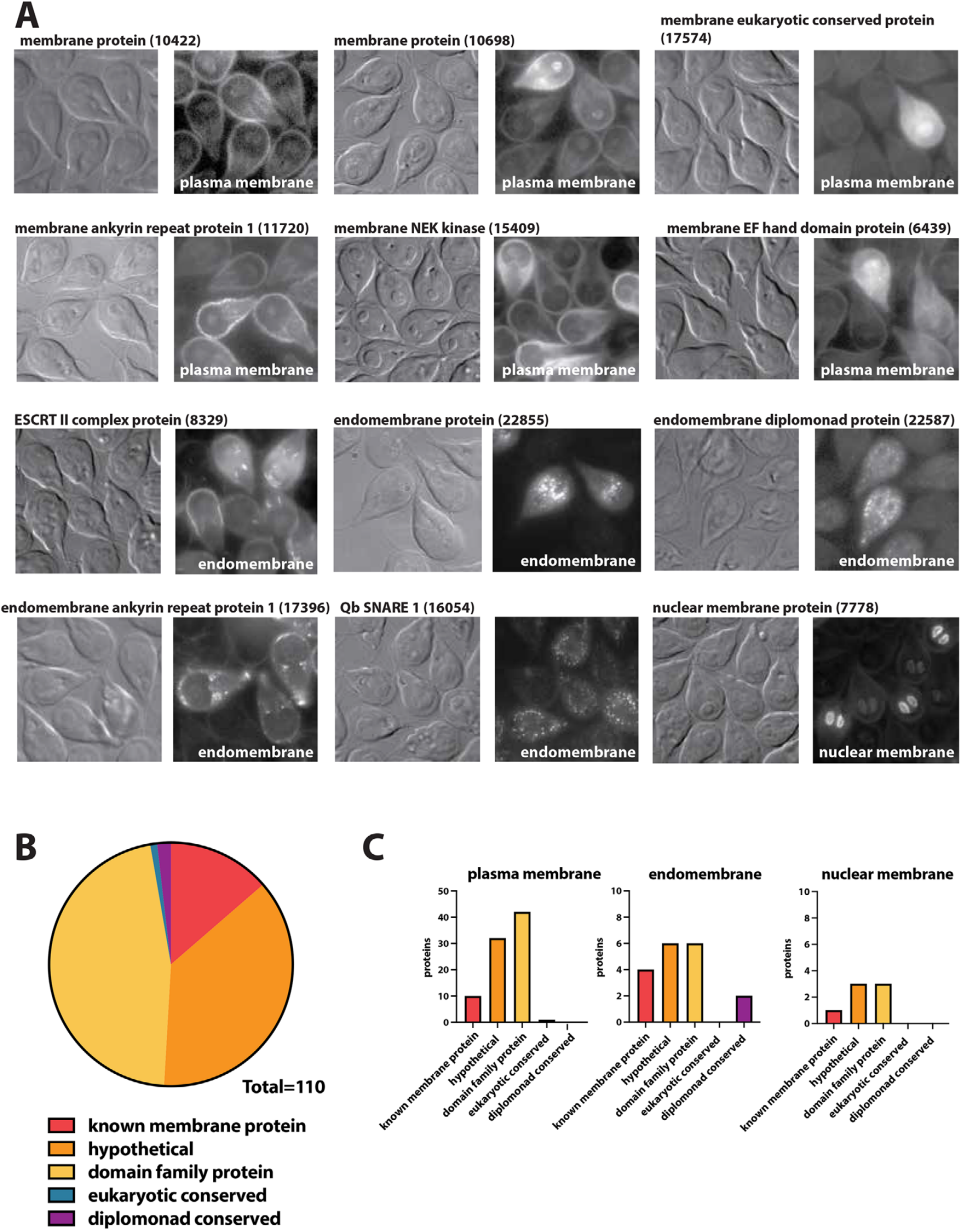
Summary localizations of plasma membrane, endomembrane systems, and nuclear membrane proteins. (A) Representative images are shown for selected tagged proteins from the 104 total proteins that localize to membrane-associated regions. GiardiaDB ORF IDs are in parentheses. (B and C) Proteins are summarized by annotation category, with “domain family proteins” including both the ankyrin repeat proteins and NEK kinase proteins.

**FIGURE 4:**
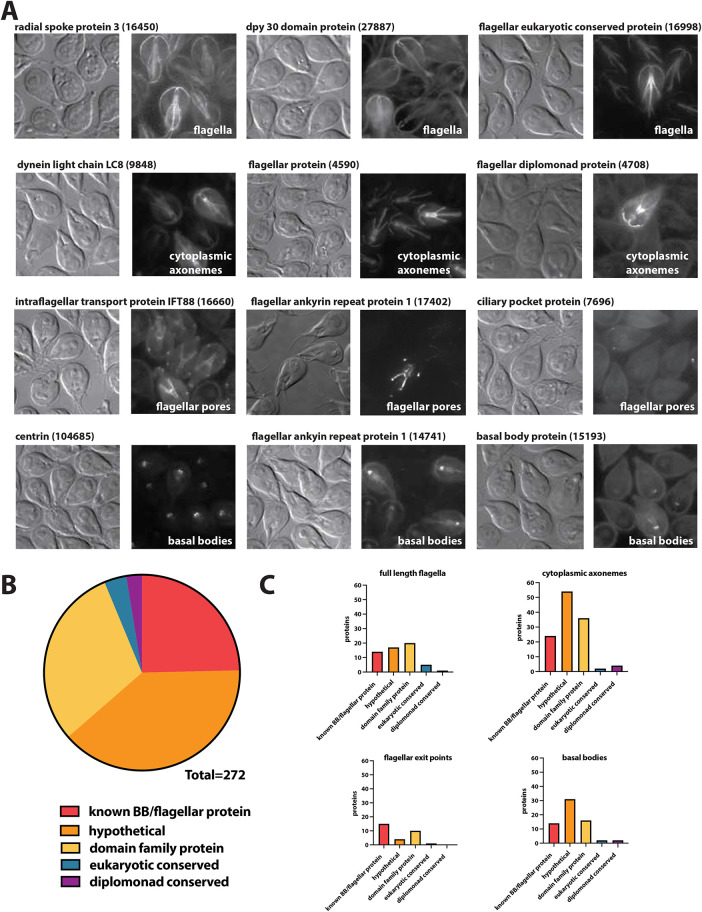
Summary localizations of flagellar and basal body proteins. (A) Representative images are shown for selected tagged proteins from the 193 total proteins that localize to the eight full-length flagella, cytoplasmic axonemes, flagellar pores at the plasma membrane–flagellar transition, and the eight basal bodies. Corresponding GiardiaDB ORF IDs are shown in parentheses. (B and C) Proteins are grouped by annotation category, with “domain family proteins” including ankyrin repeat proteins and NEK kinase proteins.

**FIGURE 5:**
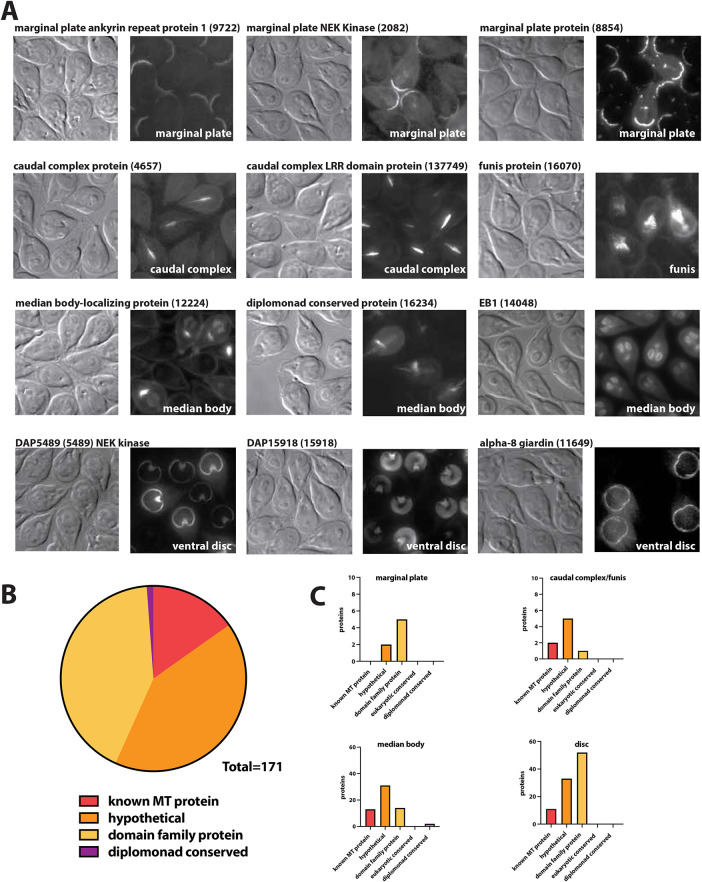
Summary localizations of proteins associated with unique *Giardia* microtubule structures. (A) Representative images are shown for selected tagged proteins from the 133 total proteins that localize to the ventral disk, median body, caudal complex, and funis, and the marginal plate. Corresponding GiardiaDB ORF IDs are indicated in parentheses. (B and C) Annotation categories are summarized, with “domain family proteins” including ankyrin repeat proteins and NEK kinase proteins.

As a positive control for localization, we tagged 77 proteins of known function, including the nucleolar protein fibrillarin (GL50803_0097219; Figure 2A), the endomembrane Qb SNARE 1 protein (GL50803_0016054; Figure 3A), the flagellar radial spoke protein 3 (GL50803_0016450; Figure 4A), and the MT-associated EB1 protein homologue (GL50803_0014048; Figure 5A). Each is localized to the relevant compartment or structure, such as fibrillarin to the nucleoli, providing a *Giardia* cellular reference marker for the other GFP-tagged protein subcellular localizations.

Many *Giardia* proteins possess conserved domains but lack other functional annotation. We focused on domain family proteins of note; thus, both ankyrin repeat proteins and NEK kinases were a functional and structural focus in this tagging project. We localized 73 ankyrin repeat proteins, representing about 25% of the 305 ankyrin repeat proteins in the 2020 reference annotation. These included *nuclear ankyrin repeat proteins* (e.g., GL50803_0014926), *membrane ankyrin repeat proteins* (GL50803_0011720), *flagellar ankyrin repeat proteins* (GL50803_0017402), *disk-associated ankyrin proteins* (GL50803_0012010) and *cytoplasmic ankyrin proteins* (GL50803_0006764) (Figures 2−4; Supplemental Table S2). The GFP localization-informed annotation also includes 47 kinases, of which 43 are NEKs (NIMA-related kinases), representing 23% of the total NEKs identified in the genome-wide dataset (184 total NEKs reported in the 2020 reference annotation). NEK kinases also localized to multiple regions, including the plasma membrane (e.g., GL50803_0015409; Figure 3A), the marginal plate (GL50803_002082; Figure 5A), and the ventral disk (GL50803_005489; Figure 5A). Beyond ankyrin repeat proteins and NEK kinases, we also tagged 99 additional domain family proteins that localized to all subcellular categories and included proteins such as a nuclear WD-40 repeat domain protein (GL50803_0015487; Figure 2A), a membrane EF hand domain protein (GL50803_006439; Figure 3A), a flagellar DPY-30 domain protein (GL50803_0027887; Figure 4A), and a caudal complex LRR domain protein (GL50803_00137749; Figure 5A).

#### New nuclear and chromosomal proteins

A total of 79 proteins were localized to the two nuclei (GO:0005634), with specific localization to regions of chromosomes or to the nucleoli, or with diffuse localization in the nuclear compartments (Figure 2, A–C). No proteins localized to only one nucleus. The majority were nuclear or chromosomal hypothetical proteins (e.g., GL50803_0013601) or proteins with conserved domains (GL50803_006367), and several nuclear proteins were conserved only in diplomonads (GL50803_00116968) (Figure 2A). Known chromatin- and transcription-related proteins localized to both nuclei, such as GL50803_0014961, a chromodomain-containing transcription factor, and GL50803_0018851, a conserved hypothetical with strong nuclear localization (Supplemental Table S2). Nine proteins that either completely lacked homology to known proteins or contained only conserved domains (e.g., nuclear ankyrin repeat protein 1 GL50803_0014926) exhibited punctate nuclear localization similar to the centromeric histone cenH3 (GL50803_0020037) (Figure 2A), suggestive of potential roles in gene regulation or chromosome structure. Finally, five proteins localized specifically to the nucleoli, including one hypothetical protein (GL50803_002340), consistent with functional roles in ribosome biogenesis or nucleolar organization.

#### Plasma membrane, endomembrane, nuclear membrane, and cytoplasmic localizing proteins

One hundred and four proteins localized to the plasma membrane, nuclear membrane, or endomembrane system, including various endocytic compartments and vesicles (Figure 3, A–C). Of these, 85 proteins localized to the plasma membrane, including hypothetical proteins (e.g., GL50803_0010422; Figure 3A), conserved proteins (GL50803_0017574), and specific domain family proteins such as a membrane ankyrin repeat protein (GL50803_0011720). Fifteen proteins localized exclusively to the endomembrane system vesicles, such as ESCRT II complex protein (GL50803_008329) or QSNARE 1 protein (GL50803_0016054), the endomembrane protein GL50803_0022855, and the diplomonad conserved protein GL50803_0022587 (Figure 3A). Seven proteins localized specifically to the nuclear envelope, including the nuclear membrane protein GL50803_007778.

A substantial number of tagged proteins (73) localized to the cytoplasm (GO:0005737, images not shown). Many cytoplasmic proteins had predicted functions such as signaling, metabolism, and molecular transport. Representative examples include GL50803_0022850, a MAP kinase (Manning *et al.*, 2011) with diffuse cytoplasmic distribution, GL50803_009909, a pyruvate phosphate dikinase, and GL50803_003861, annotated as an alcohol dehydrogenase 3. Other cytoplasmic proteins were hypothetical (e.g., GL50803_008937) or domain family proteins (e.g., GL50803_0032778, a cytoplasmic ankyrin repeat protein 1). These cytoplasmic localizations are consistent with the broad functional diversity of cytoplasmic proteins in *Giardia*, many of which remain poorly characterized despite conserved domain predictions.

#### Proteins localizing to flagella, basal bodies, and flagella pores

*Giardia* has four pairs of flagella with differing lengths, unique flagellar-associated structures, and specialized roles in motility (Dawson and House, 2010b; McInally *et al.*, 2019). Each flagellum has an internal cytoplasmic axoneme that extends from a basal body located between the two nuclei and becomes membrane-bound as it exits the cell body at the flagellar pore. One hundred and ninety three GFP-tagged proteins localized to the flagella, flagellar pores, or basal bodies (Figure 4). Because many of the 179 flagellar proteins localize to only the internal cytoplasmic portions of the flagella, we previously devised new GO terms to distinguish between the full-length flagella of each flagellar pair (anterior, caudal, posteriolateral, and ventral) and the cytoplasmic internal or membrane-bound external portions of the flagellum (Supplemental Table S1; The Gene Ontology Consortium *et al*., 2023). Sixty-nine hypothetical proteins localized to one of these regions (Figure 4A), including those specific to all flagellar regions, to just the cytoplasmic regions (e.g., GL50803_004590), or to just the anterior flagellar pores (GL50803_007696). Other conserved diplomonad proteins (GL50803_004708) or known flagellar structural proteins (radial spoke protein 3, GL5803_0016450) localize to entire flagellar structures or just to the cytoplasmic regions (flagellar ankyrin repeat protein 1, GL50803_0017402) and/or flagellar pores (intraflagellar transport protein IFT88 (GL50803_0016660).

Several proteins localized specifically to some but not all flagella, including GL50803_0015456, an ankyrin repeat protein 1 localizing only to the cytoplasmic regions of the caudal axonemes, and GL50803_0017531, a hypothetical protein that localizes only to the right anterior cytoplasmic axoneme (Supplemental Table S2).

### Localization to other cytoskeletal structures, including the ventral disk, median body, marginal plate, funis, and caudal complex

Like many other protists, *Giardia* has unique microtubule (MT) organelles such as the ventral disk and the median body. *Giardia* axonemes also have novel associated components, such as the caudal complex and funis, which are associated with the cytoplasmic regions of the caudal axonemes, and the marginal plate, a repetitive structure associated with the cytoplasmic regions of the anterior axonemes (Figure 1B). In total, 133 GFP-tagged proteins localized to one or more of *Giardia*’s unique MT organelles, and only 21 of these are previously known MT-associated proteins. Because of the novelty of these organelles, we included proteins in the GFP-tagging list derived from a total MT proteome identified from detergent-extracted MT preparations (Nosala *et al.*, 2020). Some of these represent the first proteins known to localize solely to these unique cytoskeletal structures.

Many of *Giardia’*s unique cytoskeletal structures lack known functions in *Giardia* biology (Dawson, 2010), including the flagellar- and MT-associated marginal plate, caudal complex, and funis (Figure 1B). We found seven marginal plate proteins and seven proteins localizing to the caudal complex and/or funis (Figure 5). These proteins either lack known domains (e.g., marginal plate protein GL50803_008854), or have ankyrin repeat domains (marginal plate ankyrin repeat protein 1, GL50803_009722), or NEK kinase domains (marginal plate NEK kinase GL50803_002082). We also discovered the first proteins localizing solely to the caudal complex or funis (GL50803_00137749, GL50803_004657, and GL50803_0016070).

The median body is another enigmatic *Giardia* MT organelle that is proposed to act as a reservoir to build MT structures like the ventral disk or mitotic spindles during cell division (Brugerolle, 1975; Feely, 1990; Hardin *et al.*, 2017). Here we identified 59 proteins with median body localization, including a conserved diplomonad protein (GL50803_0016234; Figure 5A). Of these, several are localized only to the median body (e.g., median body localizing protein GL50803_0012224). Other known MT-binding proteins, such as EB1 (GL50803_0014048), also localize to the median body MTs (Figure 5; Supplemental Table S2).

The ventral disk, roughly 8 µm in diameter, is perhaps the most complex *Giardia*-specific MT organelle, functioning in parasite attachment to the host gastrointestinal tract (Nosala *et al.*, 2018). Here, we have added new ventral-disk-associated proteins (or DAPs) to the inventory of known disk proteins and also include previously published DAPs (Nosala *et al.*, 2018, 2020). Ninety-three DAPs have now been characterized by subcellular localization, including 32 hypothetical proteins and 50 domain family proteins, many of which are ankyrin repeat and NEK kinase domain proteins. Only 11 previously known MT-associated proteins localize to the ventral disk. We have previously shown that some DAPs localize to specific regions of the disk such as the ventral groove or overlap zone regions (Nosala *et al.*, 2020). Many DAPs also localize to the lateral crest or disk margin (edge) regions of the disk, including ankyrin repeat domain proteins like GL50803_0017096 or annexin homologues like alpha-8 giardin (GL50803_0011649). Other DAPs, like GL50803_0015918, localize to the entire ventral disk (Figure 5A).

## REVISED LOCALIZATION-BASED PROTEIN ANNOTATIONS

Based on our extensive localization data, we provide a revised annotation—termed the “**localization-informed annotation”**—developed by integrating **subcellular localization data** from this study with **protein domain predictions** and comparative homology (Supplemental Table S2). This information was then applied to the refined genome assembly and gene models from the WB 2020 reference (Xu *et al.*, 2020). The localization-informed annotation is contrasted with prior versions from GiardaDB (Morrison *et al.*, 2007) (Alvarez-Jarreta *et al.*, 2024) and the WB2020 reference to highlight changes (Supplemental Table S2).

The revised localization-informed annotation uses a structured nomenclature that captures both protein family and localization. When domain analysis supported membership in a known family, and localization matched expected organellar distributions, annotations were updated accordingly. For instance, GL50803_0015890 is annotated as a *flagellar ankyrin repeat protein* due to its strong ankyrin repeat signal and localization to membrane-bound flagella. In contrast, GL50803_003491 is annotated as a *flagellar diplomonad conserved protein*—it lacks characterized domains or homologues outside diplomonad protists, but its flagellar localization informed the revised annotation. This annotation strategy was extended to other subcellular organelles or compartments. For instance, proteins localized to the nuclear membrane were annotated accordingly (e.g., GL50803_002340, *nuclear protein;* Figure 2A). Previously published localizations, such as the DAPs or IFT components, are indicated in the revised annotation and image dataset (Supplemental Table S2).

## SUMMARY AND SIGNIFICANCE

*Giardia* encodes many lineage-specific proteins that lack recognizable homology and likely underlie the biology of its unusual organelles, cytoskeletal architecture, and strategies for persistence in the host. In parallel, the *Giardia* genome also encodes a significant number of conserved hypothetical proteins that are shared across diverse eukaryotes but lack experimentally derived functions. Together, these complementary protein classes illuminate both *Giardia*-specific innovations and deeply conserved eukaryotic processes. Defining the biological roles of both classes of unique proteins becomes markedly more tractable once the spatial contexts of individual proteins are defined. As demonstrated here, large-scale subcellular localization anchors anonymous ORFs to defined cellular structures and pathways, transforming their previously uncertain roles into testable biological and functional hypotheses and yielding hundreds of candidate proteins for focused follow-up studies.

Thus, by systematically localizing hundreds of previously uncharacterized proteins, this study provides a powerful foothold into *Giardia*’s unique biology. The resulting revised genome annotation integrates localization data with domain architecture, homology, and *Giardia*-specific Gene Ontology terms to generate an experimentally grounded foundation for functional prediction across the proteome. Collectively, the structured nomenclature and localization-based annotations provide a lasting framework for future functional genomics in *Giardia* and related protists. When combined with ongoing multiomic analyses, this resource will advance our understanding of fundamental eukaryotic cell biology through the lens of a deeply diverged diplomonad lineage.

## Supporting information









## Abbreviations used:

APEX: enhanced ascorbate peroxidase
CRISPR: Clustered Regularly Interspaced Short Palindromic Repeats
DAP: disk associated protein
DIC: Differential Interference Contrast microscopy
FITC: Fluorescein isothiocyanate (green)
GFP: Green Fluorescent Protein
GO: gene ontology
Halo: modified haloalkane dehalogenase
MBP: median body protein
mNG: mNeonGreen
MT: microtubule
NEK: NIMA-related kinase
ORF: open reading frame
PCR: polymerase chain reaction
SNAP: Self-labeling Non-fluorescent Associated Protein
VSP: variant-specific surface protein.
