# Supplementary material for: Breaking the annotation barrier: An initial subcellular localization atlas of Giardia’s hypothetical and conserved hypothetical proteins provides a resource for functional discovery
Source: Mol Biol Cell. 2026 Apr 10;37(5):mr4. doi: 10.1091/mbc.E25-12-0590 (PMC13322205; doi:10.1091/mbc.E25-12-0590)
Supplement: Supplementary file 1 [file mbc-37-mr4-s001.pdf]

Supplemental Materials

Molecular Biology of the Cell

Hagen *et al*.

Supplemental Table 1: ***Giardia*****-specific GO annotations**

Supplemental Table 2: Revised localization-informed annotations and subcellular localizations for 608 ***Giardia*** **proteins**
